# Computation Offloading and Resource Allocation for Energy-Harvested MEC in an Ultra-Dense Network

**DOI:** 10.3390/s25061722

**Published:** 2025-03-10

**Authors:** Dedi Triyanto, I Wayan Mustika, Widyawan

**Affiliations:** 1 Department of Electrical Engineering and Information Technology, Universitas Gadjah Mada, Yogyakarta 55281, Indonesia; dedi.triyanto2481@mail.ugm.ac.id (D.T.); widyawan@ugm.ac.id (W.); 2 Department of Computer Engineering, Universitas Tanjungpura, Pontianak 78124, Indonesia

**Keywords:** mobile edge computing, computation offloading, ultra-dense network, resource allocation

## Abstract

Mobile edge computing (MEC) is a modern technique that has led to substantial progress in wireless networks. To address the challenge of efficient task implementation in resource-limited environments, this work strengthens system performance through resource allocation based on fairness and energy efficiency. Integration of energy-harvesting (EH) technology with MEC improves sustainability by optimizing the power consumption of mobile devices, which is crucial to the efficiency of task execution. The combination of MEC and an ultra-dense network (UDN) is essential in fifth-generation networks to fulfill the computing requirements of ultra-low-latency applications. In this study, issues related to computation offloading and resource allocation are addressed using the Lyapunov mixed-integer linear programming (MILP)-based optimal cost (LYMOC) technique. The optimization problem is solved using the Lyapunov drift-plus-penalty method. Subsequently, the MILP approach is employed to select the optimal offloading option while ensuring fairness-oriented resource allocation among users to improve overall system performance and user satisfaction. Unlike conventional approaches, which often overlook fairness in dense networks, the proposed method prioritizes fairness-oriented resource allocation, preventing service degradation and enhancing network efficiency. Overall, the results of simulation studies demonstrate that the LYMOC algorithm may considerably decrease the overall cost of system execution when compared with the Lyapunov–MILP-based short-distance complete local execution algorithm and the full offloading-computation method.

## 1. Introduction

Mobile edge computing (MEC) is a transformative technology that brings significant advances to wireless networks. These advances are made by maximizing the computational capabilities of edge servers embedded in mobile base stations and access points. These edge servers play a crucial role in the enhancement of the computing capabilities of mobile devices (MDs). The main advantages of MEC include its low-latency services, which are achieved by facilitating the fast response of real-time applications and minimizing the amount of data that must travel to centralized cloud servers [1]. This technology not only improves network efficiency by transferring computationally intensive tasks to edge servers, but also improves energy conservation and increases the battery life of resource-limited devices. Furthermore, MEC facilitates efficient use of network resources, reduces congestion and improves network capacity and performance in general [2]. In addition, integration of energy-harvesting technology with MEC promotes sustainability by minimizing the power consumption of MDs. In general, MEC can substantially enhance wireless networks through low-latency services, energy conservation, improved network efficiency and the integration of sustainable energy practices [3].

The computational demands of ultra-low-latency applications, such as autonomous driving and the Internet of Things, can be efficiently managed by integrating MEC with energy harvesting (EH). While MEC reduces latency by processing data at the network edge, EH ensures sustained operation of energy-constrained devices, which is particularly beneficial in fifth-generation (5G) networks [4]. MEC leverages computational resources at the network edge and facilitates fast and efficient data processing, reducing latency by offloading tasks from MDs. Thus, MEC is particularly important given the limited battery capacity of MDs [5]. The energy efficiency of the network can be further enhanced using EH techniques, ensuring the uninterrupted execution of computation-intensive tasks through relying on renewable energy sources. This integration is pivotal for supporting applications in intelligent vehicular mobile networks, such as autonomous driving and real-time video streaming, which require low-latency and high-reliability connectivity. Overall, the challenges encountered due to latency-sensitive applications are addressed by MEC with EH in 5G networks, which also promotes sustainability through green computing practices [6].

Ultra-dense network (UDN) architectures rely heavily on MEC, which provides decentralized application services and computing resources. The technical benefits of this strategy include reduced network congestion, optimized data privacy and security, and increased application speed [7]. Low-latency processing, which is essential for real-time decision-making in very dense networks, is possible through MEC. This processing is realized by placing computing power at the network edge. Moreover, transferring computation-intensive jobs from small-cell base stations to edge computing servers is possible, which reduces device stress and extends battery life [8]. MEC and UDNs work together to improve the computing capabilities of MDs while simultaneously expanding the network capacity. The implementation of this architecture necessitates a system model that considers the very dense deployment of edge servers and base stations. An optimization problem should also be established to determine the best resource-allocation plan and computation-offloading technique [9]. Overall, the installation of edge computing servers within small-cell base stations is necessary for the MEC design of UDNs to provide low-latency processing and realize the offloading of computation-intensive tasks.

This work investigates the difficulties related to computation offloading and resource allocation in MEC within the setting of UDN. The study looks at how limited computing resources and task delays affect system performance, with a focus on how to best offload computations and distribute resources. The key contributions can be encapsulated as follows:

- The UDN is built on an MEC architecture integrated with EH technology. A task-execution cost model that accounts for execution latency and energy consumption is developed. The integration of task offloading with resource allocation is characterized as an NP-hard optimization issue.
- The challenges related to computation offloading and resource allocation are resolved using the LYMOC approach. This technique combines the Lyapunov algorithm with mixed-integer linear programming (MILP)-based optimum cost. The Lyapunov algorithm was selected due to its ability to successfully handle the dynamic and stochastic characteristics of MEC settings by stabilizing the energy queue, hence guaranteeing efficient energy use over a period of time. MILP was used due to its ability to efficiently address the intricate resource-allocation issue through determining the appropriate method of distributing jobs from many mobile devices to various MEC servers.
- LYMOC effectively addresses the need for real-time resource allocation in high-user-density networks. This method ensures fairness-oriented allocation, balancing resource distribution among users in dense environments, preventing service degradation and improving overall network efficiency.

The subsequent sections of this document are organized in the following manner: Section 2 provides a comprehensive analysis of the existing research in the area of MEC and UDNs. Section 3 introduces the system model and outlines the problem formulation. Section 4 provides a comprehensive explanation of the proposed LYMOC approach. Section 5 presents the experimental results and offers a comparison analysis with the baseline algorithms. Section 6 provides a discussion, concludes the work and proposes options for further research.

## 2. Related Work

The integration of MEC with UDN optimizes network velocity, reduces latency and facilitates efficient deployment of advanced services through the use of distributed processing capabilities and multiple small cells. The deployment of MEC in UDNs to enhance the performance of 5G networks is examined in [7]. The proposed MEC architecture provides a system model within the UDN and formulates optimization problems. This study uses the action-classification (AC) method, particularly the DQN-AC algorithm, to identify the most effective solutions for computation offloading and resource allocation. Similarly, a system model for an MEC-assisted UDN is presented in [10], with the aim of minimizing the system overhead. The proposed methodology decomposes the problem into smaller components, including offloading strategies, channel assignments and power distribution. Joint offloading and resource-allocation algorithms enable the implementation of optimal solutions. The challenges posed by the increasing number of intelligent terminal devices in 5G, AI and IoT environments are addressed by a dense edge computing system that integrates MEC with UDN, as discussed in [11]. The challenges related to task offloading and resource scheduling in UDNs are explored in [12], where MEC is used to minimize overall system costs, including latency and energy consumption. Solutions for task offloading, base station selection and resource scheduling for mobile devices are also proposed. Furthermore, ref. [13] introduces a cost-efficient hierarchical approach (HACO) to improve computation offloading and resource allocation in ultra-dense multi-cell MEC networks. This method reduces financial expenditures and addresses non-convex challenges using the Artificial Fish Swarm Algorithm (IAFSA) and Particle Swarm Optimization (IPSO).

The integration of computation offloading and resource allocation in MEC systems aims to reduce latency by intelligently selecting offloaded tasks and efficiently allocating resources, thus optimizing system performance. By leveraging edge servers for intensive processing, this strategy can effectively reduce energy consumption and enhance application responsiveness. Deep reinforcement learning (DRL) is applied in [14] to provide energy-efficient task offloading, optimizing rewards while considering task deadline constraints. Additionally, DRL methods are employed in various offloading scenarios. The study in [15] utilizes deep Q-learning for decision-making in distributed offloading while considering edge node load dynamics. Another approach to leveraging distributed DRL is introduced in HOODIE [16], which implements hybrid offloading in Cloud-Edge frameworks to improve resource allocation in dynamic latency conditions. Researchers in [17] demonstrate that the Stackelberg game framework is an effective approach for multiple agents to collaboratively optimize the trade-off between latency and energy efficiency in vehicular networks. A novel stochastic game-based computation-offloading model and a multi-agent reinforcement learning algorithm, namely SGRA-PER, are proposed in [18], exhibiting superior performance in dynamic resource allocation.

Trajectory control for MEC is also explored in various studies. Two trajectory-control algorithms are introduced in [19], consisting of a convex optimization-based trajectory-control algorithm and a DRL-based trajectory-control algorithm. The Lyapunov-based multi-agent deep deterministic policy gradient technique, which jointly optimizes task distribution and radio resource allocation, is proposed in [20]. A task-offloading optimization strategy is presented in [21], employing Lyapunov optimization and a DRL-based resource-allocation approach. However, a major limitation of conventional DRL methods is their slow adaptation to environmental changes. To overcome this challenge, the study in [22] introduces DMRO, a meta-reinforcement learning framework designed to accelerate decision-making in dynamic environments. This method enhances performance by integrating DRL with meta-learning, enabling faster model adaptation in MEC environments. A novel approach called JVFRS-CO-RA-MADDPG, based on DRL, is proposed in [23], which optimizes video frame resolution, computation offloading and resource allocation collectively. Additionally, a comprehensive framework is developed in [24] to manage task delegation, resource allocation and trajectory planning for cooperative edge computing among multiple UAVs, emphasizing the importance of task prioritization in optimizing system performance.

Several studies have examined the trade-off between execution latency and energy consumption to enhance MEC efficiency. The study in [25] explores task-offloading strategies in wireless-powered MEC networks (WP-MEC), which integrate wireless energy transfer and offload optimization to minimize energy consumption. This model employs a two-stage multi-agent deep reinforcement learning-based distributed computation-offloading (TMADO) framework to optimize task distribution and maintain low energy costs. The efficient management of computational activities in MEC systems enhances resource utilization, reduces operational costs and mitigates network congestion. The WiDaS method, described in [26], utilizes Lyapunov optimization and the water-filling concept for task scheduling, aiming to minimize average task-response time while adhering to resource constraints. The stochastic computation-offloading problem is analyzed in [27], where a virtual queueing technique based on Lyapunov optimization is implemented to ensure system stability. Our preliminary research [28] contributes to this discussion by analyzing energy efficiency based on data-transmission distance, data rate, CPU frequency and transmission power, and by investigating energy trade-off in computing offloading. The DAEE method is proposed for adaptive offloading, with the goal of maintaining low latency while minimizing long-term energy consumption. An online resource-offloading and -allocation technique, based on Lyapunov optimization, is introduced in [29], effectively addressing dynamic computational demands and user mobility while balancing energy efficiency and service latency. A cost-minimization offloading model is formulated in [30], optimizing edge computing resources, signal-detection vectors, transmission power and IRS coefficients to achieve a balance between latency and energy consumption. A mixed optimization problem is described in [31], where the authors propose PATA and DHC methods to efficiently address the energy-latency trade-off by optimizing power allocation, task scheduling and clustering mechanisms.

This research distinguishes itself from previous studies by incorporating energy-harvesting technology into MEC and improving the efficiency of task offloading and resource allocation in UDNs. The primary objective is to enhance sustainability and reduce mobile device power consumption, particularly in 5G networks requiring ultra-low latency and high reliability. This study differs from prior research by evaluating system performance in highly dense UDN scenarios and employing a more realistic energy-harvesting model. Furthermore, it emphasizes fairness-oriented resource allocation to ensure equitable resource distribution among users in UDN environments, which is critical for maintaining user satisfaction and balanced system performance.

This paper provides significant advantages compared to prior studies, as shown in Table 1, especially in terms of integrating energy-collection technology with MEC. This connection enhances sustainability by decreasing the power consumption of mobile devices. The LYMOC technique, which is an optimization approach, successfully deals with the dynamic and stochastic characteristics of the MEC setup by stabilizing the energy queue, enhancing energy efficiency and achieving fairness in resource allocation. In addition, the system model takes into account UDN situations that involve numerous MD and MEC servers. It also addresses the specific issues linked to interference and coordination among devices. The cost function in this optimization issue incorporates both execution delays and penalties for completed jobs, thereby guaranteeing optimization of performance, fairness and dependability.

## 3. System Model and Problem Formulation

This section provides an explanation of the system model and problem formulation for our research on computing offloading and resource allocation in MEC combined with EH in a UDN. MEC has become an essential technology to address the growing need for high processing power and fast response times in current applications, especially in the context of 5G networks and beyond. Integrating EH technologies with MEC improves sustainability through decreasing the dependence on conventional power sources and prolonging the lifespan of MDs.

### 3.1. System Model

**Figure 1:**
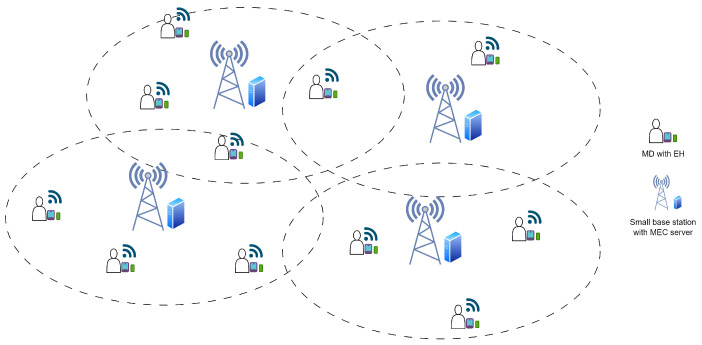
MEC architecture under the UDN scenario.

This study explores an MEC architecture within a UDN, as illustrated in Figure 1. The system comprises a set of MDs, denoted as M={1,2,3,…,M},each equipped with EH capabilities. Concurrently, a small base station (SBS) equipped with an MEC server exists. A set of MEC servers, which are denoted as S={1,2,3,…,S}, provide essential computing resources and services. The suggested system model takes into account the distinctive attributes of UDN, including the reduced distance between devices and increased interference. When there are a lot of devices and base stations in UDN, the environment is very complex and changes dynamically. As a result, interference among devices becomes severe. It is important for devices and MECs to collaborate to complete the task. Hence, this system model considers the unique attributes of UDN, such as precise device and server spacing and rigorous interference management. Additionally, the communication infrastructure employs orthogonal frequency division multiple access (OFDMA), which establishes connections among users, communication channels and MEC servers. In this system, each MD is allocated a dedicated communication channel to the MEC server and prevents direct task collisions at the transmission level. However, if multiple tasks are transferred simultaneously to MEC servers, processing delays may occur due to resource conflicts. The work does not include additional scheduling mechanisms on the MEC server to resolve these conflicts, which can be considered in future improvements. OFDMA facilitates efficient frequency resource allocation by dividing the frequency channel into subchannels, each assignable to a specific user. This algorithm enables simultaneous and effective communication between MDs and MEC in the UDN. Each MD is assumed to traverse the SBS area at a specific distance, which is quantified using the Euclidean distance formula.

Within this framework, the temporal dimension of the MEC system is organized into slots, *t*, where T={1,2,3,…,T} represents its index set. In addition, each MD-based application initiates a time slot with a probability of success, ρ, and failure, with a probability of 1−ρ. This condition indicates that computation tasks follow an independent and identically distributed (i.i.d.) Bernoulli process, contributing to the dynamic and probabilistic nature of the system. Each MD is tasked with computing-intensive operations that can be executed either locally on the MD itself or offloaded to the MEC servers for processing. Additionally, neither of the two calculation modes is feasible; for example, if the energy supply of the MD is inadequate, then the computation task will be dropped. Thus, the computation mode of MD indicates the time frame as $\alpha_{m}+\alpha_{s}+\alpha_{d}=1$, where $\alpha_{m}=1,\alpha_{s}=1,\alpha_{d}=1$ denote the execution of the processing task at the MD, offloading or dropping it to the MEC server. The edge server has unlimited computational capabilities, resulting in minimal execution time. The feedback time is disregarded due to the substantial disparity in size between the outcome and the raw data [32]. This assumption is based on the fact that the results of the computation task are much smaller than the input data, and in most scenarios the transfer delay of the returned results is negligible.

The parameters and notations used in this study are summarized in Table 2.

### 3.2. Communication Model

The system model comprises several SBSs and MDs in the UDN scenario. Each MD has a job that requires considerable computing power that can be sent to a single MEC server over the wireless channel. The feasible offloading-transmission rate can be computed as follows, assuming that the system employs OFDMA techniques [33]:

(1) $$R_{m,s}\left(t\right)=\omega \log_{2}\left(1+\frac{H_{m,s}\left(t\right)P_{m,s}\left(t\right)}{\sigma^{2}}\right)\;\forall m\in M,\forall s\in S$$

where ω is the bandwidth of the wireless channel, $P_{m,s}\left(t\right)$ is the MD transmission power for data offloading, $\sigma^{2}$ is the white Gaussian noise that occurs during data transmission and $H_{m,s}\left(t\right)$ is the wireless channel gain [7].

To mitigate severe inter-cell interference caused by the high density of MDs and SBSs in UDNs, co-channel deployment is not utilized. Instead, OFDMA is employed to assign orthogonal sub-channels to each MD, ensuring interference-free communication and more stable transmission rates, albeit at the cost of reduced spectral efficiency.

The wireless channel gain $H_{m,s}\left(t\right)$ is represented by:

(2) $$H_{m,s}\left(t\right)=A_{g}{\left(\frac{3\times 10^{8}}{4\pi f_{c}r}\right)}^{\zeta }\;\forall m\in M,\forall s\in S$$

where ζ is the path loss exponent, *r* is the distance between the MD and MEC servers, $f_{c}$ is the carrier frequency and $A_{g}$ is the antenna gain. Therefore, if the calculation task is performed by the MEC server, then the execution delay is equivalent to the transmission delay for the input bits, $L_{m}$.

(3) $$D_{m,s}\left(t\right)=\frac{L_{m}\left(t\right)}{R_{m,s}\left(t\right)}\;\forall m\in M,\forall s\in S.$$

Consequently, considering energy consumption, the energy used for transmission can be computed as follows [32]:

(4) $$E_{m,s}\left(t\right)=P_{m,s}\left(t\right)D_{m,s}\left(t\right)\;\forall m\in M,\forall s\in S$$

The transmission power is assumed to be limited to address practical considerations; that is, $P_{m,s}\left(t\right)\leq P_{m,s}^{max}\left(t\right)$.

### 3.3. Local Computing Model

The energy usage and time delay in local execution depend on the resources of the MD. The execution of applications and local management of tasks are realized by using the computational resources available on the MD, such as its CPU and memory. The energy consumption of local computing in the MD can be stated as follows [32]:

(5) $$E_{m}\left(t\right)=k_{m}L_{m}c{\left(f_{m}\left(t\right)\right)}^{2}\;\forall m\in M.$$

Furthermore, the local computing task-execution time can be determined in accordance with

(6) $$D_{m}\left(t\right)=\frac{L_{m}c}{f_{m}\left(t\right)}\;\forall m\in M$$

where $k_{m}$ is the effective switched capacitance determined by the chip architecture, *c* is the number of CPU cycles necessary for processing a single-bit input and $f_{m}\left(t\right)$ is the CPU cycle frequencies that are planned for local execution during time slot *t*. The top limit of the CPU-cycle frequency of any MD is denoted as $f_{m}^{max}$; that is, $f_{m}\left(t\right)\leq f_{m}^{max}\left(t\right)$.

### 3.4. Energy Model

This EH model considers factors such as the availability and intensity of renewable energy sources and the efficiency of the energy conversion process. The model helps determine the amount of energy that can be stored in the battery of an MD based on the assumption of uniformly distributed harvestable energy $E_{H}\left(t\right)$ with a maximum value $E_{H}^{max}\left(t\right)$. The assumption of uniformly distributed harvested energy $E_{H}\left(t\right)$ is employed as a simplifying assumption to enhance the problem’s tractability [34]. While dynamic energy variations are typically captured using linear or nonlinear energy-harvesting models, this approach focuses on a statistical representation that facilitates optimization. A portion of the energy that occurs during each time slot, designated as e(t) and satisfying $0\leq e\left(t\right)\leq E_{H}\left(t\right)$, is collected, stored in a battery and made accessible for local execution or computation offloading at the next time slot. Furthermore, the model considers the energy consumption of the device and its battery storage capacity. Let E(t) represent the energy spent by the MD during time slot *t*. The energy consumption is dependent on the chosen computation method, which is represented as follows:

(7) $$E\left(t\right)=\alpha_{m}E_{m}\left(t\right)+\alpha_{n}E_{m,s}\left(t\right)\;\forall m\in M,\forall s\in S$$

The energy usage during each time slot must not exceed the level of the battery; that is, E(t)≤B(t). Therefore, the battery energy level changes in accordance with the following formula:

(8) B(t+1)=B(t)−E(t)+e(t)∀t∈T.

The formula actually ensures that the battery level stays within the range of feasible scope at all times. Since each time slot energy consumption is limited by E(t)≤B(t), the battery level is updated based on available energy and harvested energy and prevents overflow and violation of physical storage limitations.

### 3.5. Problem Formulation

The optimization problem in the setting of UDN exhibits numerous fundamental distinctions when compared to typical MEC. The large number of devices and base stations in UDN increases interference, and therefore there is a need for effective resource allocation. Thus, this problem formulation takes into account the distinct limitations encountered in a densely populated setting, such as effectively handling interference and promoting improved coordination among devices. The proposed Lyapunov–MILP optimization technique is specifically developed to efficiently tackle the unique issues of UDN. It ensures proper resource allocation and computation offloading to minimize both delay and energy usage. Given these complexities, optimization problems are combinatorial due to decisions on the allocation of binary tasks and continuous resource constraints, making them mixed integer optimization problems. These characteristics require the use of an optimization method that can efficiently handle both discrete and continuous decision variables, which is why MILP is selected for this problem.

The execution cost function comprises two components: the time required to execute the task and the penalty associated with dropping or not completing the activity, expressed as

(9) $$Z\left(t\right)=\sum_{m=1}^{M}\sum_{s=1}^{S}\left(\alpha_{m}D_{m}+\alpha_{s}\beta_{m,s}D_{m,s}\right)+\gamma \cdot 1\left(\rho =1,\alpha_{d}=1\right)$$

where $\beta_{m,s}$ denotes the decision to pair each MD with MEC containing the least cost, and γ is the weight of the task-dropping cost. $\alpha_{m}$ and $\alpha_{s}$ are dimensionless coefficients that equilibrate the effects of $D_{m}$ and $D_{m,s}$, whereas $\beta_{m,s}$ is a binary variable devoid of dimensions that signifies the connection decision between a mobile device and a server. Simultaneously, γ denotes the penalty weight for omitted tasks, articulated in seconds, in accordance with the time units of $D_{m}$ and $D_{m,s}$.

Identifying the optimal option for offloading and allocating resources accordingly is necessary to reduce the overall cost of the system. The current issue is delineated in the following manner:

(10) $$\begin{array}{cc} P1 & :\min_{\alpha_{c},P_{m,s},f_{m},\beta_{m,s}}\lim_{T\to \infty }\frac{1}{T}E\left[\sum_{t=0}^{T-1}Z\left(t\right)\right]\\ \; & s.t.\\ C1 & :\alpha_{c}\left(t\right)\in \left\{0,1\right\},c\in \left\{\alpha_{m},\alpha_{s},\alpha_{d}\right\},\forall_{t}\in T\\ C2 & :0\leq f_{m}\left(t\right)\leq f_{m}^{max}\left(t\right),\forall_{t}\in T,\forall_{m}\in M\\ C3 & :0\leq P_{m,s}\left(t\right)\leq P_{m,s}^{max}\left(t\right),\forall_{t}\in T,\forall_{m}\in M,\forall_{s}\in S\\ C4 & :0\leq e\left(t\right)\leq E_{H}\left(t\right),\forall_{t}\in T\\ C5 & :E\left(t\right)\leq B\left(t\right),\forall_{t}\in T\\ C6 & :\beta_{m,s}\leq U^{max},\forall_{t}\in T,\forall_{m}\in M,\forall_{s}\in S\\ C7 & :\beta_{m,s}\geq 1,\forall_{t}\in T,\forall_{m}\in M,\forall_{s}\in S\\ C8 & :\beta_{m,s}\in \left\{0,1\right\},\forall_{t}\in T,\forall_{m}\in M,\forall_{s}\in S \end{array}$$

where *C1* represents the zero-one indicator restriction of the computation mode indicators. The ranges of the MD CPU cycle frequency and transmission power values are denoted by *C2* and *C3*, respectively. The MD obtains energy in the form of *C4*. The conversion loss prevents the energy obtained during the current period from exceeding its value. *C5* ensures that the computing task’s energy consumption does not exceed the remaining energy available during time slot *t*. Constraint *C6* assures that the cumulative demand on a server does not exceed its maximum capacity, $U^{max}$, while *C7* guarantees that every mobile device establishes a connection with at least one server, facilitating job execution. *C8* explicitly designates $\beta_{m,s}$ as a binary variable to signify the connection decision between a mobile device and a server. These constraints function at different levels—*C6* operates at the server level to regulate resource capacity, whereas *C7*–*C8* manage individual device connectivity—ensuring that the system operates in harmony without conflict.

## 4. Proposed Method

Two methodologies are utilized to tackle the task-offloading and resource-allocation issues in UDN-based multi-user MEC systems with EH devices, as delineated in the problem specification. The Lyapunov drift-plus-penalty method is employed to dynamically optimize offloading decisions, reducing execution costs while accounting for task failures and execution delays. Second, the MILP method is used to make the best use of each MD’s resources at every time slot. This makes sure that computational tasks are assigned to MEC servers efficiently while still meeting capacity limits. The method also makes the best use of transmission power and computational resources to lower the cost of execution.

The MILP method is selected because of its effective management of both binary and continuous decision variables, rendering it ideal for optimizing task offloading and resource allocation in UDN. The binary nature of the decision to execute tasks locally or remotely, contrasted with the continuous nature of resource allocations like transmission power and CPU frequency, makes MILP an effective optimization framework that guarantees feasibility within the constraints of execution time, energy consumption and server constraints (*C6-C8*). While DRL may be utilized to address P1, it necessitates long training and meticulous hyperparameter optimization, and does not consistently ensure optimal solutions in dynamic environments [35]. Conversely, MILP ensures global optimality for structured issues such as P1, rendering it especially beneficial for optimizing task-to-server assignments and resource allocation in UDN, where deterministic solutions are preferred over exploration-based learning [36]. The suggested method uses MILP to make sure that resources are used efficiently, fair task distribution and execution costs are kept low. This makes it a strong and reliable choice for task offloading in high-density MEC settings.

### 4.1. Lyapunov Algorithm

The perturbation parameter was initially established, and the virtual energy line at the MD was constructed. Perturbation parameters are used to alleviate the existing energy causality limitations inside the optimized dynamical system, thus enabling the estimation of the upper limit of energy consumption for local execution and offloading [6]. The perturbation parameter, ϵ, is expressed as

(11) $$\epsilon \geq {\tilde{E}}_{max}+\frac{V\cdot \gamma }{E_{min}}$$

where ${\tilde{E}}_{max}=\min\left\{\max\left\{k_{m}L_{m}c{\left(f_{m}\right)}^{2},p_{max}T_{d}\right\},E_{max}\right\}$ and *V* is a control parameter (in $J^{2}s^{-1}$).

A displaced representation of the actual battery energy level on an MD is referred to as the concept of a virtual energy queue. The virtual energy queue, which serves as a metric for reducing the energy consumption from the mobile battery and optimizing the use of renewable energy sources, is quantified as follows:

(12) $$\tilde{B}\left(t\right)=B\left(t\right)-\epsilon .$$

This method ensures adherence to constraint *C5*. This constraint controls energy causality by making sure that the energy used for local computation and offloading does not go over the energy that is available in the MD’s battery at any given time. The virtual energy queue and the perturbation parameter are used by the system to dynamically control the amount of energy used, which makes operations more sustainable and efficient.

Lyapunov theory is used to ensure the stability of the queue. Initially, the quadratic sum of the queue backlog is computed as follows:

(13) $$\begin{array}{c} L\left(t\right)=\frac{1}{2}\sum_{m=1}^{M}{\left(\tilde{B}\left(t\right)\right)}^{2}\\ L\left(t+1\right)=\frac{1}{2}\sum_{m=1}^{M}{\left(\tilde{B}\left(t+1\right)\right)}^{2}. \end{array}$$

Thus, the Lyapunov drift function can be mathematically represented as

(14) $$\Delta \left(t\right)=E\left[L\left(t+1\right)-L\left(t\right)|\tilde{B}\left(t\right)\right]$$

and the expression for the Lyapunov drift-plus-penalty function is

(15) $$\Delta_{V}\left(t\right)=\Delta \left(t\right)+V\cdot E\left[Z\left(t\right)|\tilde{B}\left(t\right)\right].$$

Lyapunov’s theorem indicates that the stability of the virtual energy queue and the finiteness of the queue backlog is guaranteed when the above equation is satisfied.

(16) $$\lim_{T\to \infty }\frac{\tilde{B}\left(t\right)}{T}=0.$$

According to (16), Problem P1 may be reformulated as Problem P2, which incorporates minimal drift-plus-penalty.

(17) $$\begin{array}{cc} P2 & :\min_{\alpha_{c},P_{m,s},f_{m},\beta_{m,s}}\sum_{t=0}^{T-1}\tilde{B}\left(t\right)\left(e(t)-E(t)\right)+V\cdot E\left[Z\left(t\right)|\tilde{B}\left(t\right)\right]\\ \; & s.t.\;C1–C4,C6–C8. \end{array}$$

This reformulation is conditioned on all constraints in P1. This reformulation is unrelated to the stability and dependability of the system. Thus, the second optimum objective in the deterministic problem P2 mentioned above will be temporarily disregarded. In addition, the second optimum objective is directly addable to P2.

### 4.2. Optimal Computation Offloading

The best amount of harvested energy can be determined by solving a linear programming problem that reduces the amount of energy harvested from the battery while maintaining a non-negative virtual energy queue.

(18) $$\min_{0\leq e\left(t\right)\leq E_{H}\left(t\right)}\tilde{B}\left(t\right)e\left(t\right).$$

The best EH choice of each MD is independent of others. Thus, the optimal e(t) may be calculated individually for each MD. This equation effectively addresses constraint *C4* by ensuring that the harvested energy does not exceed the available battery energy and maintains a non-negative virtual energy queue.

In relation to the computing modes, obtaining the corresponding mode to the smallest $G_{CO}$ value for every individual MD is necessary. The formula in [6] allows for the calculation of the optimal CPU cycle frequencies, $f_{m}^{*}$, and optimal transmission power, $P_{m,s}^{*}$.

The optimization of $f_{m}$, the CPU-cycle frequency for local execution, is performed by minimizing the cost function, which includes both energy consumption and execution delay. The cost function $G_{M}$ is defined as:

(19) $$\begin{array}{cc} \min_{f_{m}} & \;-\tilde{B}\left(t\right)k_{m}L_{m}\left(t\right)c{\left(f_{m}\left(t\right)\right)}^{2}+V\cdot \frac{L_{m}\left(t\right)c}{f_{m}\left(t\right)}\\ s.t. & \;C2. \end{array}$$

The optimal frequency $f_{m}^{*}$ is determined by setting the first-order derivative of $G_{M}$ to zero, which results in $f_{m}^{*}={\left|\frac{V}{2\cdot -\tilde{B}\left(t\right)\cdot k}\right|}^{\frac{1}{3}}$.

The optimization of $P_{m,s}$, the transmission power for offloading to the MEC server, is performed by minimizing the cost function, which includes both energy consumption and communication delay. The cost function $G_{S}$ is defined as:

(20) $$\begin{array}{cc} \min_{P_{m,s}} & \;-\tilde{B}\left(t\right)\frac{P_{m,s}L_{m}\left(t\right)}{R_{m,s}\left(t\right)}+V\cdot \frac{L_{m}\left(t\right)}{R_{m,s}\left(t\right)}\\ s.t. & \;C3. \end{array}$$

The first-order derivative of $G_{S}$, solved using the Newton-Raphson method and set to zero, is used to determine the optimal transmission power $P_{m,s}^{*}$: $\tilde{B}\left(t\right)\cdot \log_{2}\left(1+\frac{H_{m,s}\left(t\right)P_{m,s}\left(t\right)}{\sigma^{2}}\right)$$+\frac{H_{m,s}\left(t\right)\cdot \left(V\;-\;\tilde{B}\left(t\right)\cdot P_{m,s}\left(t\right)\right)}{\ln\left(2\right)\cdot (\sigma^{2}\;+\;H_{m,s}\left(t\right)P_{m,s}\left(t\right))}=0.$

The optimal objective may be realized using P2, as indicated by the following formula:

(21) $$G_{CO}=\alpha_{m}\cdot G_{M}\left(f_{m}^{*}\left(t\right)\right)+\alpha_{s}\cdot \beta_{m,s}\cdot G_{S}\left(P_{m,s}^{*}\left(t\right)\right)+\alpha_{d}\cdot V\cdot \gamma$$

where $G_{M}$ and $G_{S}$, which are subproblems for the local and remote execution modes, respectively, have $G_{M}\left(f_{m}^{*}\left(t\right)\right)$ and $G_{S}\left(P_{m,s}^{*}\left(t\right)\right)$ as their corresponding optimum objectives. The optimal values of $G_{CO}$ for the three computing modes can be analyzed to determine the optimal computation-offloading choice, which can be defined as follows:

(22) $$\alpha_{c}=\arg\min_{\alpha ,f_{m},P_{m,s}}G_{CO}.$$

### 4.3. Resource Allocation

The alignment of MD and MEC servers—with the objective of optimizing resource allocation and minimizing the geographical distance between the edge server and MD—is realized utilizing the MILP approach. This approach considers several characteristics, including the execution cost and the server load, to identify the most appropriate server. The decision to pair each MD with one of the MEC servers, $\beta_{m,s}$, may be obtained using P3 to resolve the allocation of MD task resources to a server.

(23) $$\begin{array}{cc} P3 & :\min_{\beta_{m,s}}\sum_{m=1}^{M}\sum_{s=1}^{S}\beta_{m,s}{\left(D_{m,s}\right)}^{T}\\ \; & s.t.\;C6–C8. \end{array}$$

The objective is to reduce the overall cost while adhering to limitations *C6*, *C7* and *C8*. The MILP strategy incorporates the selection of the server with the lowest cost as the decision variable while also considering the constraints of maximum server capacity and the need for each MD to be connected to at least one server. The MILP issue is resolved by the use of a conventional optimization solver. This solver systematically explores possible combinations of $\beta_{m,s}$ while adhering to all constraints in order to locate the optimum solution. The solver utilizes branch-and-bound methods and cutting planes to effectively explore the solution space, guaranteeing the selection of the most suitable server for each MD.

### 4.4. LYMOC Algorithm

The specific implementation process is described in Algorithm 1 The LYMOC method efficiently manages task allocation and resource utilization in MEC inside UDNs by dynamically optimizing computation offloading for MDs. At first, the characteristics and placements of MDs and MEC servers are established. MD positions are randomly updated for each time slot. The method computes the distance between MDs and MEC servers, modifies the gathered energy if necessary and verifies task requests. When a task request is present, the system calculates the local execution latency and energy consumption. It then assesses the transmission delay and energy consumption for each server and selects the most suitable server using a MILP method. The selection of the appropriate computing mode is based on the generalized computing offloading, $G_{CO}$, value and the execution cost, energy consumption and chosen mode are recorded. Ultimately, the battery level is refreshed, and the cycle is repeated for the subsequent time interval.
**Algorithm 1:** LYMOC Algorithm
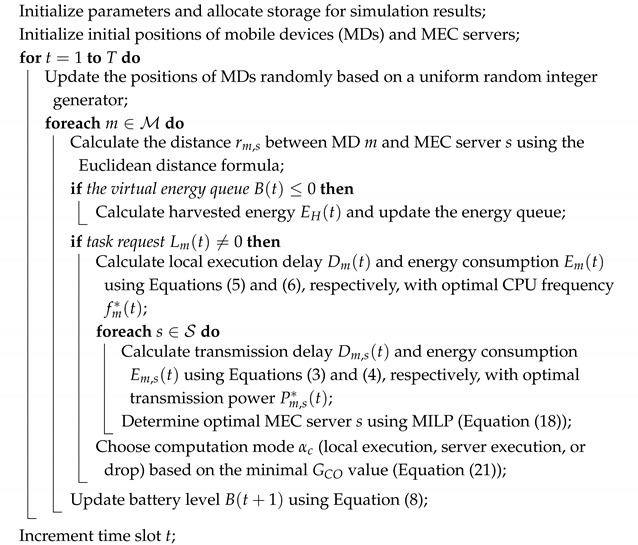


### 4.5. LYMOC Algorithm Computational Complexity

The LYMOC algorithm’s computational complexity is assessed in the manner described below. Time slots *T*, mobile devices (MDs) *M* and MEC servers *S* are present. The LYMOC algorithm’s system input complexity is O(TMS), meaning that the number of time slots, mobile devices and MEC servers all cause the input size to rise linearly. In this case, the complexity of resource allocation using the MILP method is $O(2^{M\cdot S})$, which is exponential in the number of mobile devices (*M*) and MEC servers (*S*). Thus, $O(T\cdot M\cdot \left(S+2^{M\cdot S}\right))$ is the total complexity of the LYMOC method, which integrates time complexity, input size and resource allocation using MILP. This demonstrates that the total complexity grows linearly with the number of time slots (*T*) and exponentially with the number of mobile devices (*M*) and MEC servers (*S*). The MILP-based LYMOC algorithm offers an optimum solution for resource allocation in MEC systems, resulting in a substantial reduction in execution costs when compared to other approaches. Although there is a greater computational burden, the advantages in terms of energy efficiency and the time it takes to complete tasks make it worthwhile to utilize.

## 5. Experimental Results and Analysis

### 5.1. Convergence Analysis

Convergence analysis is crucial to ensuring that the proposed algorithm achieves stable and optimal performance under dynamic and stochastic conditions in UDNs. In this section, we analyze the convergence behavior of the proposed algorithm both theoretically and empirically, focusing on key metrics such as the average battery energy level, execution cost and energy consumption.

#### 5.1.1. Theoretical Convergence Guarantee

The theoretical foundation of the proposed algorithm is based on Lyapunov optimization. The stability of the algorithm is guaranteed by minimizing the Lyapunov drift-plus-penalty function, which combines system stability with optimization of task execution and resource allocation.

The Lyapunov drift Δ(t) represents the variation in the Lyapunov function L(t) across consecutive time intervals, as expressed in Equation (14). The Lyapunov function L(t) is expressed in Equation (13), and $\hat{B}\left(t\right)=B\left(t\right)-\theta$ indicates the energy level in the virtual battery following the application of the perturbation parameter θ. To keep things stable, the method reduces the drift-plus-penalty function: Equation (15), where Z(t) is the optimization target and includes execution latency, energy use and penalties for quitting a task. The control parameter V>0 delineates the balance between system stability and optimization efficacy.

The stability of the virtual battery queue is guaranteed by satisfying the following Lyapunov condition: $\lim_{T\to \infty }\frac{1}{T}\sum_{t=0}^{T-1}E\left[\hat{B}\left(t\right)\right]<\infty$. This condition ensures that the queue backlog remains bounded, demonstrating that the algorithm converges to a stable state under dynamic and stochastic conditions.

#### 5.1.2. Empirical Validation

Empirical results from simulations further validate the theoretical convergence guarantees. In this study, Table 3 shows the system-simulation parameters that were used. These include bandwidth, transmission power and CPU and network characteristics. The selected parameter values are utilized to represent actual conditions and guarantee the accuracy of the simulation outcomes.

**Figure 2:**
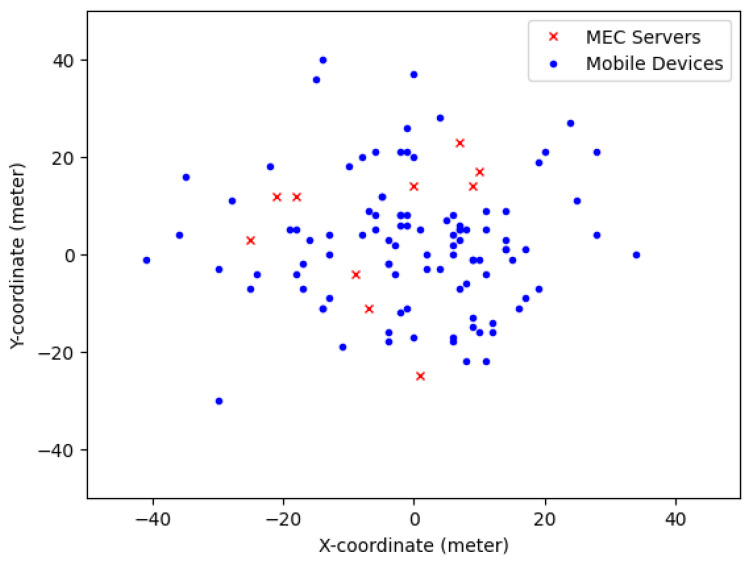
Simulation of MD movement against MEC servers.

We established a high-density network simulation scenario that included 10 SBSs with MEC capabilities, covering an area of 10,000 m^2^, and distributed 100 MDs throughout this region. Each MD was outfitted with energy-harvesting functionalities, with energy availability approximated probabilistically. We constructed computation jobs using a binomial distribution, formulating tasks based on a specific probability. In this simulation, the initial locations of MDs and MEC servers were randomly assigned within designated ranges, with MDs positioned inside a 10-m by 10-m region centered at (0,0) and MEC servers situated within a 50-m by 50-m area around the origin. The system revised the position of each MD at each *t* time interval, using a step length to enable random movement in any direction. The Euclidean distance between each MD and MEC server was computed following each movement update. Figure 2 illustrates the distribution of MEC servers (represented by red symbols ‘x’) and MDs (represented by blue dots ‘o’) within the simulation region after the 200th time frame.

**Figure 3:**
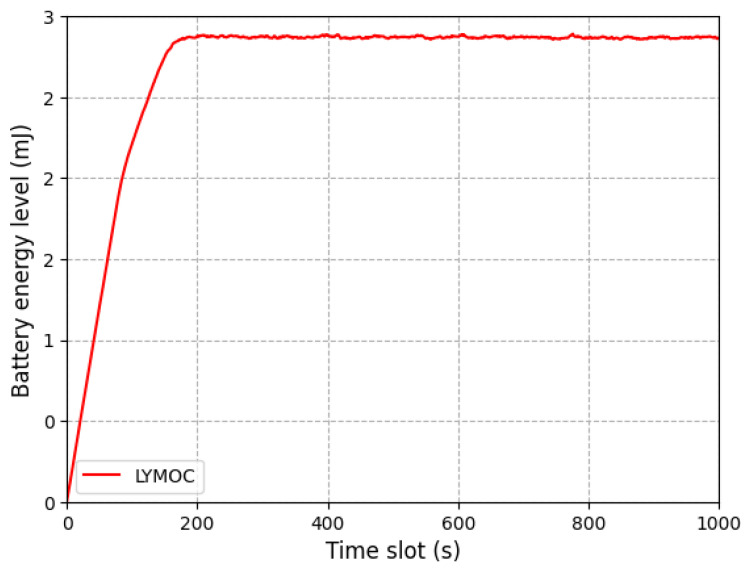
Battery energy level.

The battery energy level represents the amount of accumulated energy in the battery of an MD at a specific moment. Figure 3 illustrates the convergence of the battery energy level over time under the proposed method. The energy level dynamically changes based on the MD’s energy usage, which is influenced by the chosen computing mode, the planned CPU cycle frequency and the assigned transmit power. The battery energy level adheres to an energy causality constraint, ensuring that the MD’s energy consumption does not exceed the available energy in the battery. If the battery energy level is insufficient to complete a computational task, the task is either terminated or dropped. This constraint ensures that the remaining energy can be utilized for other activities or to extend the battery’s lifespan. The proposed method demonstrates stable convergence in maintaining the battery energy level over time. This result highlights the effectiveness of energy management, which balances computing and transmission energy expenditures. The stability and convergence are achieved by minimizing the Lyapunov drift-plus-penalty function, as discussed in Section 5.1.1.

### 5.2. Performance Comparison and Scalability

The study looked at how well the LYMOC algorithm worked compared to three other algorithms: Lyapunov–MILP-based shortest distance (LYMSD), local execution all (LEA) and offloading all (OA). LEA presumed that all users favored local computing, OA presumed that all users favored edge computing and LYMSD allocated resources depending on the minimal distance between MDs and MEC servers.

**Figure 4:**
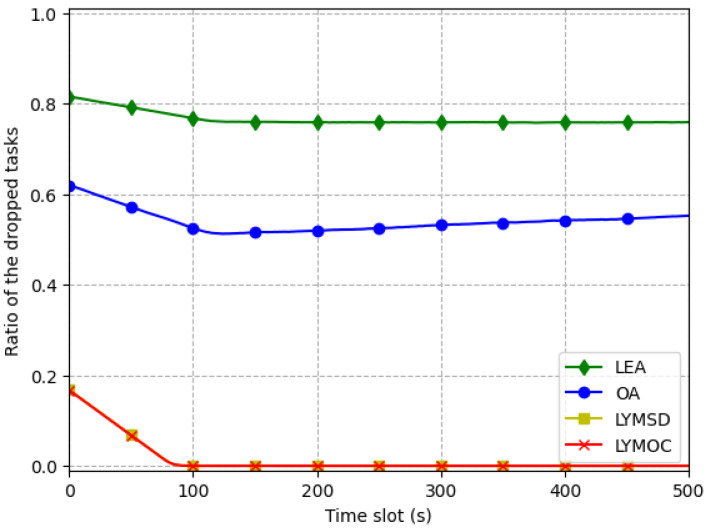
Task-drop ratio comparison.

Efficient energy management is crucial to avoiding task failures. Figure 4 shows that LYMOC and LYMSD have the lowest task-drop rates, followed closely by OA and LEA. The overall performance of the system is optimized by the dynamic task-allocation capabilities of LYMOC and LYMSD. The impairment ratio of the LYMOC and LYMSD methods is only slightly different (0.084 and 0.085, respectively), but LEA has the largest ratio of lost jobs. This phenomenon occurs because LEA abstains from assigning tasks to edge servers, resulting in task interruption or postponement when available power is insufficient to perform the ongoing task. OA outperforms LEA in terms of the lost task ratio by transferring all tasks to edge servers, thereby optimizing performance. However, this method might lead to high expenses due to the considerable energy used for transmitting tasks to the server.

**Figure 5:**
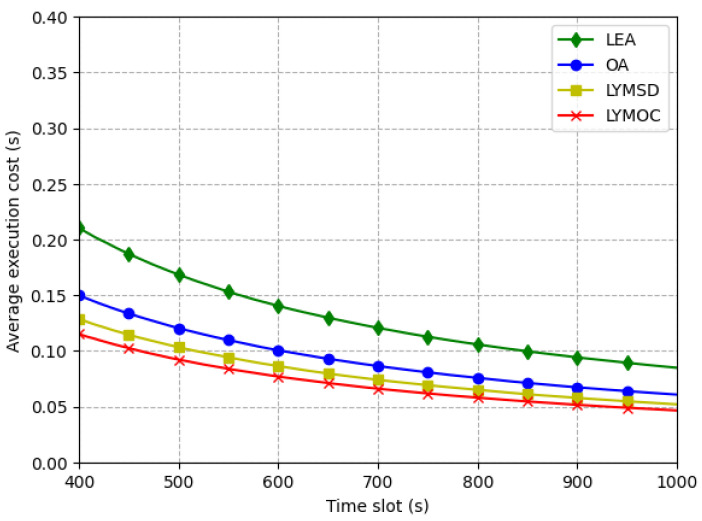
Average execution cost per time slot.

Figure 5 depicts the average execution cost for each method and emphasizes the efficiency ratio of LYMOC in relation to LYMSD, LEA and OA. The LYMOC algorithm exhibits the lowest execution cost, surpassing LYMSD, LEA and OA by optimizing resource consumption and reducing system load. This cost-effectiveness improves system responsiveness, facilitating swifter reactions to user requests or activities while conserving energy and extending battery life for MDs. Furthermore, LYMOC’s efficiency ratio enhances over time, indicating its exceptional potential to maximize work performance over prolonged periods. A one-way analysis of variance (ANOVA) was used to prove these results statistically. The results showed that the algorithms were significantly different (F-statistic: 11.172, $p=2.68\times 10^{-7}$). A post-hoc Tukey HSD test verified that LYMOC and LYMSD greatly surpassed LEA in minimizing execution costs. Nonetheless, no substantial differences were detected among LYMOC, LYMSD and OA, suggesting equivalent performance under particular conditions. The results underscore the LYMOC algorithm’s efficacy in minimizing execution costs, especially in comparison to the LEA.

**Figure 6:**
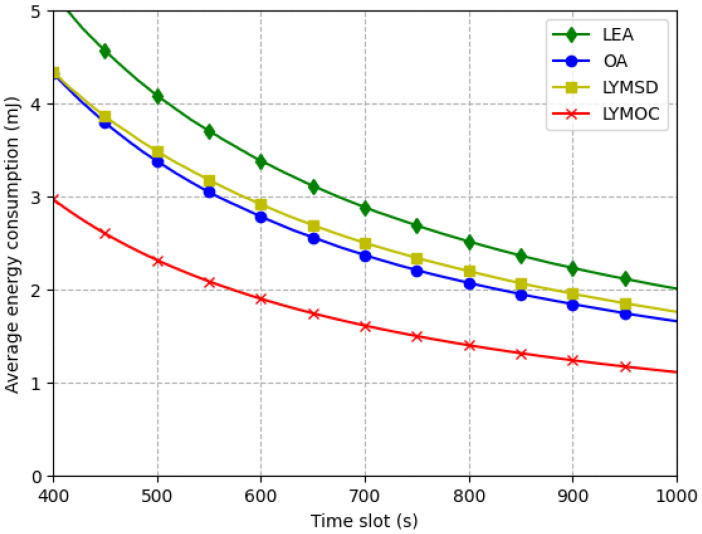
Average energy consumption per time slot.

Figure 6 depicts the diminishing average energy consumption of the four algorithms as the time interval extends. The OA algorithm allocates all work to the edge, potentially necessitating significant energy, especially when the edge server is distant. Conversely, the LEA method does all calculations locally on the device, resulting in substantial energy consumption when the device possesses constrained processing capabilities. The LYMSD algorithm enhances energy efficiency by employing the most direct path between devices and base stations, hence minimizing energy usage, particularly when devices are in close proximity. The LYMOC method exhibits optimal energy efficiency through the optimization of computation-offloading and resource-allocation strategies. By dynamically picking the most energy-efficient offloading mechanism and execution time, LYMOC optimizes resource allocation by selecting suitable servers to reduce energy usage. With an F-statistic of 3.542 and a *p*-value of 0.014 (p<0.05), the ANOVA test shows that the algorithms use statistically different amounts of energy on average. Post-hoc Tukey HSD tests reveal significant disparities among algorithm pairs, demonstrating that LYMOC consistently attains the lowest energy usage, hence validating its exceptional energy efficiency. In contrast, LEA demonstrates the greatest energy usage attributable to inefficiencies in resource allocation. The results underscore the efficacy of LYMOC in optimizing energy use, rendering it a more energy-efficient option for the evaluated scenario.

**Figure 7:**
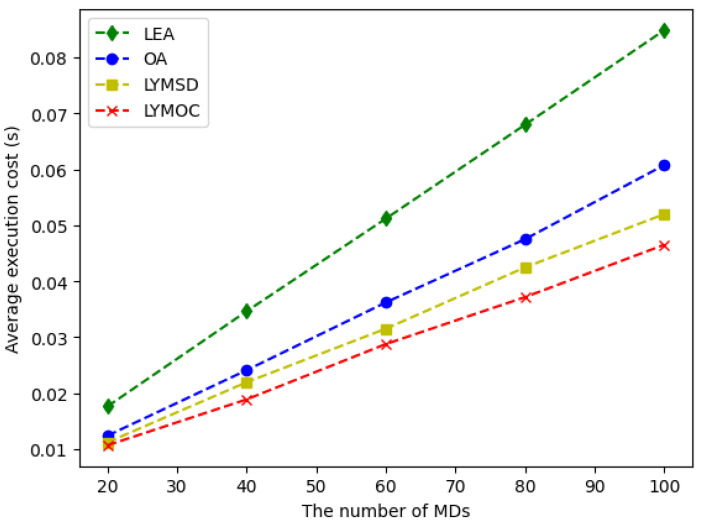
The impact of the number of MDs on the average execution cost.

Finally, Figure 7 depicts the average execution cost of four different methodologies for task execution on an MEC system: LEA, OA, LYMSD and LYMOC. A regression analysis was conducted for each approach to examine the correlation between the number of MDs and the average execution cost. The linear regression model was utilized to assess the strength of the link, with $R^{2}$ values reflecting the quality of fit for each method. All approaches exhibit a substantial correlation between the quantity of MDs and the average execution cost, with $R^{2}$ values surpassing 0.999, so affirming a robust linear relationship. Since LEA runs all jobs locally on MDs, which causes congestion and performance issues as the number of devices grows, it has the most noticeable linear increase in execution cost (slope: 0.000839). The execution costs of OA grow in a logarithmic way because all jobs are sent to a remote edge server. This means that as the number of MDs grows, so do the costs of bandwidth and latency. LYMSD surpasses OA by utilizing efficient offloading strategies that reduce communication expenses linked to task transfer to the server. LYMOC, exhibiting the minimal slope (0.000450), attains sublogarithmic rise in execution cost by dynamically optimizing task execution using Lyapunov optimization and employing MILP for effective resource allocation. This hybrid methodology equilibrates local execution and offloading, guaranteeing negligible increases in execution costs while preserving scalability. LYMOC exhibits the most advantageous balance between performance and cost, underscoring its exceptional efficiency and agility in managing the increasing volume of MDs.

The LYMOC algorithm was evaluated against three other algorithms: LEA, OA and LYMSD. The test findings indicate that LYMOC continuously maintains superior battery energy levels, exhibits the lowest task-drop ratio and achieves the lowest average execution cost when compared to LEA, OA and LYMSD. LYMOC exhibited higher energy efficiency and scalability as the number of devices rose, giving it a more dependable and adaptable solution for MEC systems in UDNs. The key performance measures utilized encompass execution cost, energy efficiency, task-drop ratio and scalability, all of which validate the dominance of LYMOC in task management and resource optimization for this scenario.

## 6. Discussion, Conclusions and Future Directions

### 6.1. Discussion

The results of the experiment indicate that the LYMOC algorithm exhibits superior performance compared to the baseline methods in many settings, such as high-density and energy-harvesting environments. The adaptive methodology of LYMOC for offloading computing and allocating resources results in significant improvements in execution cost and energy efficiency, hence decreasing task-drop rates and preserving greater battery levels in mobile devices. The results validate the durability and ability of LYMOC to expand, emphasizing its capacity to improve the efficiency of MEC systems in highly populated network environments for the tested scenarios. Notwithstanding these encouraging outcomes, the investigation has several constraints. One of the key limitations is that the current model does not explicitly address tasks queues and schedules on the MEC server, which could affect task-execution delays under high workload conditions. In addition, systems assume a dedicated communication channel per MD, but interference and congestion in the context of practical network scenarios can still affect performance. These aspects should be further analysed in future work to improve the practical application of the proposed approach. The simulations may not accurately replicate real-world intricacies, such as unexpected user conduct and fluctuating ambient conditions. In addition, the energy-harvesting paradigm pre-supposes a steady energy acquisition, which may not always be feasible in practical applications.

### 6.2. Conclusions

The results of this study show that the LYMOC algorithm makes it much easier to decide how to offload tasks and divide up resources in MEC systems within UDNs. Combining Lyapunov optimization with mixed-integer linear programming makes the LYMOC method a good way to cut down on execution costs and energy use. It possesses the capability to adjust to evolving network conditions. The experimental results confirm that LYMOC outperforms existing methods, including LEA, OA and LYMSD, regarding execution cost, energy efficiency and task-completion rate under the evaluated conditions. According to the results, LYMOC can improve the performance and efficiency of MEC systems. This makes it a reliable and flexible choice for situations that need fast processing in 5G applications.

### 6.3. Future Directions

Further investigations should prioritize the validation of the LYMOC algorithm in practical applications to evaluate its effectiveness in a wider range of varied and uncertain circumstances. It would be interesting to investigate the incorporation of advanced energy-harvesting models and adaptive algorithms capable of managing sudden changes in network circumstances. Furthermore, exploring the influence of security and privacy issues on the decision-making process of offloading computations in MEC systems is a potential and encouraging avenue for future research. Moreover, future research will also look at multi-tier offloading strategies where MEC servers decide whether to treat tasks locally or forward them to cloud servers, depending on system load and latency constraints. The improvement of MEC server system adaptability scheduling policies also improves performance in dense network environments. Finally, the integration of real-time energy-harvesting variables into the model would make it more robust in dealing with practical deployment challenges. Through addressing these problems, the usefulness and robustness of the LYMOC algorithm in actual contexts would be substantially improved.

## Figures and Tables

**Table 1:** A comparative analysis of various work.

Ref.	EH	Multi User	Multi Edge Server	Cloud Server	Optimization Objectives	Methods
[7]	-	🗸	🗸	-	Minimizing execution time, energy consumption	Reinforcement Learning (DQN-AC)
[10]	-	🗸	🗸	-	Minimization execution delay and energy consumption	MILP + Lagrangian Dual Method
[11]	🗸	🗸	🗸	-	Maximizing computation efficiency, balancing energy consumption	Residual Energy-Based Computation Efficiency (RECE)
[13]	-	🗸	🗸	-	Minimizing monetary cost under delay constraints	Hierarchical Algorithm (HACO: IAFSA + IPSO)
[18]	-	🗸	🗸	-	Minimization energy computation and delay execution	Stochastic games
[19]	-	🗸	🗸	🗸	Minimization energy computation	DRL-based dynamic trajectory control
[20]	-	🗸	🗸	-	Minimization energy computation and queue stability	Lyapunov + Multi-Agent Deep Deterministic Policy Gradient (L-MADDPG)
[21]	-	🗸	🗸	-	Minimizing energy consumption and latency	Lyapunov + DRL
[27]	-	🗸	-	-	Minimizing energy consumption and latency	Lyapunov optimization
[30]	-	🗸	🗸	-	Minimizing energy consumption and latency	Lyapunov + Stochastic Optimization
[31]	-	🗸	-	🗸	Energy efficiency, delay tradeoff	SCA algorithm
[16]	-	🗸	🗸	🗸	Latency minimization, hybrid task offloading	Distributed DRL
[22]	-	🗸	🗸	🗸	Dynamic offloading with fast adaptation	Meta Reinforcement Learning
[25]	🗸	🗸	🗸	-	Energy provision minimization	Multi-Agent Deep Reinforcement Learning (TMADO)
Our Work	🗸	🗸	🗸	-	Minimization execution delay and energy consumption	Lyapunov + MILP (LYMOC)

**Table 2:** List of notation used in this study.

Notation	Definition
M	Set of mobile devices
*M*	Number of mobile devices (M=|M|)
S	Set of MEC servers
*S*	Number of MEC servers (S=|S|)
T	Set of time slots
*T*	Number of time slots (T=|T|)
$k_{m}$	The effective switched capacitance that depends on the CPU chip architecture
*c*	The number of CPU cycles needed on processing one bit of task
ω	The bandwidth of the wireless channel
$\sigma^{2}$	White Gaussian noise
$A_{g}$	The antenna gain
$f_{c}$	The carrier frequency
*r*	The distance between the MD and MEC servers
ζ	The path loss exponent
$f_{m}\left(t\right)$	The scheduled CPU cycle frequencies for local computing
$L_{m}\left(t\right)$	The input size of the computation task
$R_{m,s}\left(t\right)$	The data-transmission rate between MD *m* and MEC servers *s*
$H_{m,s}\left(t\right)$	The wireless channel gain between MD *m* and MEC servers *s*
$P_{m,s}\left(t\right)$	The transmission power for data offloading
$D_{m,s}\left(t\right)$	The transmission delay
$E_{m,s}\left(t\right)$	The energy consumption for transmission
$E_{m}\left(t\right)$	The local computing’s energy consumption
$D_{m}\left(t\right)$	The local computing task-execution time
B(t)	The battery energy level

**Table 3:** The system-simulation parameters.

Parameters	Values	Parameters	Values
ω	1 MHz	$k_{m}$	$10^{-28}$
$\sigma^{2}$	−130 dBm	$L_{m}$	1 kB
$T_{d}$	2 mS	*c*	737.5 cycles/bit
$A_{g}$	4	γ	2 mS
$f_{c}$	0.915 GHz	$E_{max}$	2 mJ
ζ	3	$E_{min}$	0.02 mJ
*V*	$10^{-5}$ J^2^$s^{-1}$	$P_{m,s}^{max}$	1 W
$U^{max}$	10	$f_{m}^{max}$	1.5 GHz

## Data Availability

Data is contained within the article.

## Abbreviations

The following abbreviations are used in this manuscript:

MEC	Mobile edge computing
UDN	Ultra-dense network
MD	Mobile device
SBS	Small base station
LEA	Local execution all
OA	Offloading all
LYMOC	Lyapunov–MILP-based optimal cost
LYMSD	Lyapunov–MILP-based short distance
